# Postbiotics as Adjuvant Therapy in Cancer Care

**DOI:** 10.3390/nu16152400

**Published:** 2024-07-24

**Authors:** Vyshnavy Balendra, Roberto Rosenfeld, Chiara Amoroso, Cecilia Castagnone, Maria Grazia Rossino, Ornella Garrone, Michele Ghidini

**Affiliations:** 1 Saint James School of Medicine, Park Ridge, IL 60068, USA; veeb023@gmail.com; 2 Oncology Unit, Fondazione IRCCS Cà Granda Ospedale Maggiore Policlinico, 20122 Milan, Italy; roberto.rosenfeld@policlinico.mi.it (R.R.); mariagrazia.rossino@policlinico.mi.it (M.G.R.); ornella.garrone@policlinico.mi.it (O.G.); 3 Gastroenterology and Endoscopy Unit, Fondazione IRCCS Cà Granda Ospedale Maggiore Policlinico, 20122 Milan, Italy; chiara.amoroso@policlinico.mi.it; 4 National Order of Biologist, 10122 Turin, Italy; ceciliacastagnone.nutrizionista@gmail.com

**Keywords:** postbiotics, cancer, inflammation, immunity, gut microbiome

## Abstract

Postbiotics are defined as a preparation of inanimate microorganisms and/or their components that confers a health benefit to the host. They range from cell wall fragments to metabolites, bacterial lysates, extracellular vesicles, and short-chain fatty acids (SCFAs). Postbiotics may influence carcinogenesis via a variety of mechanisms. They can promote homeostatic immune responses, reduce inflammation, induce selective cytotoxicity against tumor cells, as well as the enabling the control of tumor cell proliferation and enhancing intestinal epithelial barrier function. Therefore, probiotics can serve as an adjunct strategy in anticancer treatment together with chemotherapy and immunotherapy. Up to now, the only relevant postbiotics used as interventions in oncological patients remain vitamin K molecules, with few phase-II and III trials available. In fact, postbiotics’ levels are strictly dependent on the gut microbiota’s composition, which may vary between individuals and can be altered under different physiological and pathological conditions. Therefore, the lack of consistent clinical evidence supporting postbiotics’ efficacy is due to their poor bioavailability, short half-life, and fluctuating levels. Synbiotics, a mixture of prebiotics and probiotics, are expected to have a more homogeneous bioavailability with respect to postbiotics and may have greater potential for future development. In this review, we focus on the role of postbiotics as an adjuvant therapy in cancer treatment.

## 1. Introduction

The human gut is home to millions of bacteria and is termed the “gut microbiota” in reference to this diverse population of organisms [1]. Within the gastrointestinal tract, the large intestine is the most heavily colonized by bacteria, with 500 different types of anaerobic bacteria. This human organ contains 10^11^–10^12^ bacterial cells per gram [2]. The interplay of trillions of bacterial, viral, and fungal components allows the gut microbiome to remain in homeostasis, which is pivotal to the function of the human body, the regulation of energy, and the body’s overall well-being [3]. Apart from digestion and nutrient absorption, the microbiome of the gut plays a pivotal role in supporting general health and well-being. This is maintained through a balance of microbiota which increases the integrity of the gut barrier and strengthens the immune response [4]. Subsequently, robust immunity prevents against pathogen invasion.

Dysbiosis, or the dysregulation of the gut microbiota, is the perturbation in function, composition, and lack of diversity in the microbiome [5]. This disequilibrium can lead to chronic inflammation and impact gut permeability, resulting in increased susceptibility to health conditions such as type 2 diabetes, irritable bowel disease, Parkinson’s disease, and cardiovascular diseases [6]. Alterations made in the gut microbiota are due to diet, lifestyle changes, genetics, psychological states, prebiotics, pharmaceutical treatments, and postbiotics [7].

Specifically, postbiotics are products with a low molecular weight which are fragments resulting from the fermentation process of intestinal live bacteria. Postbiotics are believed to contribute to various health benefits similar to probiotics, such as supporting gut health, modulating the immune system, and potentially influencing metabolic processes [8,9]. In comparison to probiotics, which include live microorganisms, postbiotics do not contain live bacteria. They also have clear chemical structures, safety dose regulations, and a long shelf life, providing the required stability for use in certain food products and supplements [10]. Research has shown that postbiotics have effective absorption, metabolism, and excretion features, indicating their high capacity to signal different organs and tissues in the host, thus eliciting several biological and physiological responses [11].

Recently, evidence has shown that postbiotics have the ability to strengthen and fortify gut microbiomes, and potentially serve as an oncological therapeutic plan [12]. In this review, we synthesize the collected data to provide a coherent analysis conveying an understanding of postbiotics, their features, and their potential benefits as an adjuvant therapy in the management of cancer patients.

## 2. Materials and Methods

The authors conducted an electronic search across the PubMed, Medline, C Google Scholar, and Embase library databases for peer-reviewed articles and reviews published after the year 2000. The following MeSH terms were used: probiotics, cancer, postbiotics, microbiome, cancer therapy, short-chain fatty acids, extra-cellular vesicles, AND models. Case reports were excluded. The results were further screened by title and abstract for studies performed in rodents and humans, at which time full-text articles in English language were screened for eligibility.

## 3. Prebiotics, Probiotics, and Postbiotics: An Overview

In the realm of gut health and overall well-being, prebiotics, probiotics, and postbiotics play crucial roles [13]. These terms are often used interchangeably, but even though all three can be taken through the diet or supplementation, they represent distinct concepts with diverse impacts on human health.

Prebiotics are substances that the human digestive system is unable to digest and which are therefore, when remaining in the intestinal lumen, metabolized by the beneficial bacteria of the intestine and promote their growth and activity [14]. Essentially, they serve as food for probiotics, helping them to thrive and carry out their beneficial functions [15]. Common prebiotics include nondigestible polysaccharides such as inulin, oligosaccharides, fructooligosaccharides, and galactooligosaccharides [16]. The healthiest, most common and easiest way to take prebiotics is through the diet. Only in some cases where, due to specific pathological conditions, the individual is unable to satisfy his fiber needs may supplements be used [17]. Prebiotics can be useful for keeping the intestinal microbiota healthy and therefore regulating digestion, and also for supporting the immune system [18]. Prebiotics, however, are not recommended in cases of IBS (irritable bowel syndrome) and in cases of lactose intolerance [19]. Probiotics are live microorganisms, typically strains of bacteria or yeasts, that manage to reach the intestine still alive and active, and they offer health benefits as they create a balance in the intestinal microflora when consumed in adequate amounts [20]. Common probiotics include Lactobacillus and Bifidobacterium. They are found in fermented foods and in supplement form [21].

Probiotics can be easily consumed in food, making sure to include fermented foods such as yogurt and kefir in the diet [22]. Natural food is always preferable to taking probiotics, but in cases where this is not possible or after and during antibiotic therapies which are very harmful to the microbiota, it is recommended to take them via supplements [23]. Probiotics can help to restore the balance of the intestinal flora which can be altered due to unbalanced diets, drug therapies, or certain types of pathologies. Their consumption can improve digestion [24], strengthen the immune system [25], and even positively influence mental health [26]. They have no contraindications for those suffering from irritable bowel syndrome or lactose intolerance; on the contrary, they can help to reduce the symptoms of these problems [27].

Postbiotics are a preparation of inanimate microorganisms and/or their components that confers a health benefit to the host [8,28]. These may include substances such as organic acids, enzymes, peptides, and polysaccharides that are produced during the fermentation process. Postbiotics are present in fermented foods but can also be taken via supplements. They can offer similar benefits to probiotics but without the need to introduce live microorganisms into the gut [29].

In conclusion, while prebiotics, probiotics, and postbiotics work together to promote gut health, each has a unique role and offers distinct benefits, as described in Table 1. Incorporating all three into the diet can help to maintain a healthy balance of the gut flora and promote overall well-being.

## 4. Types of Postbiotics

**Figure 1:**
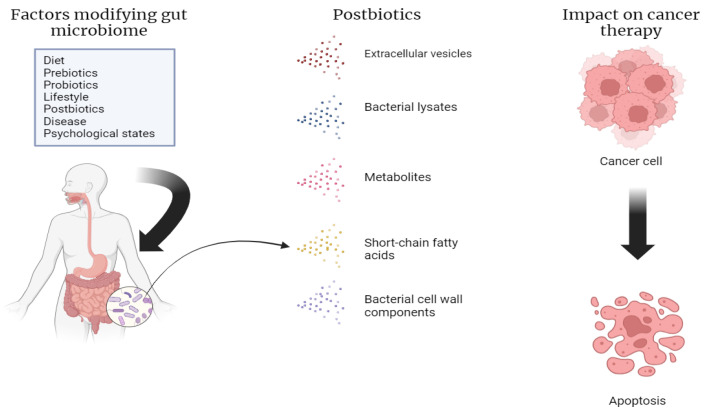
Factors that modify the gut microbiome, including the various types of postbiotics. Our own elaboration based on the data in [8,11,12,13]. This figure was created using Biorender.com (accessed on 1 July 2024).

Postbiotics range from cell wall fragments to metabolites, bacterial lysates, extracellular vesicles, and short-chain fatty acids (SCFAs). They are classified by their chemical composition, origin, and functional properties [30] (Figure 1).

**Short-Chain Fatty Acids (SCFAs):** SCFAs are organic acids with a carbon chain length of six carbons or less [31]. They are the main metabolites created by intestinal bacteria during the fermentation of plant polysaccharides. Common SCFAs include acetic, propionic, and butyric acids, which can form the fatty acid salts acetate, propionate, and butyrate, respectively [32]. SCFAs are vital in sustaining and maintaining gut health, regulating immune responses, and metabolism. Specifically, butyrate is an energy source used mainly by enterocytes to regenerate and revive the intestinal epithelium, and has also been shown to have immunosuppressive characteristics [33]. This specific SCFA also regulates gene expression through the suppression of histone deacetylases [34].

**Bacterial Cell Wall Components:** Bacterial cell wall components include the components of bacterial cells such as peptidoglycans, lipopolysaccharides cell surface proteins, and nucleic acids which interact with the host immune system to regulate immune responses [35]. Bacterial lipoteichoic acid (LTA) is an immunogenic component of the cell walls of Gram-positive bacteria [36]. LTA has demonstrated immunostimulatory properties such as inducing a reduction in IL12 production and the production of cytokines with immunoregulatory activity [37]. Studies have shown LTA to be beneficial in treating skin infections. The topical use of LTA increases barrier defense mechanisms and the release of peptides such as human β-defensin and cathelicidin prevents infections [38].

**Bacterial lysates (BLs):** BLs are soluble substances that are released during the degradation of Gram-positive and Gram-negative bacteria during bacterial cell lysis [39]. Lysates have been beneficial in decreased inflammatory diseases like ulcerative colitis and Crohn’s disease [40]. They replenish the gut microbiome, strengthen intestinal barrier integrity, control immune responses, regulate immune cells functions, and decrease the growth of pathogens [41].

**Metabolites:** The gut microbiota consists of a variety of molecules such as vitamins, enzymes, and bioactive compounds. Vitamins, in particular, have a high bioavailability with antioxidant properties which aid in host–microbome interaction [42]. Folate is taken up by the colon and is vital for physiological processes and is incorporated in the host tissue for DNA replication, repair and methylation [43]. Intestinally produced folate delivers a beneficial systemic function. Studies have shown those living in countries with fortification folate in their foods had a decreased risk of stroke compared to the controls [44]. Enzymes also possess defense mechanisms in protecting proteins, nucleic acids, and lipids against oxidative stress [45]. Antioxidant enzymes such as superoxide dismutase, glutathione peroxidase, NADH-oxidase, peroxide dismutase, and catalase help to protect organs and tissues from reactive oxygen species. Lactobacillus lactis is a postbiotic enzyme expresses catalase which has been shown to inhibit the metastasis of colon cancer, while *L. plantarum* postbiotics have been observed to increase concentrations of glutathione peroxidase in serum [46].

**Extracellular vesicles (EVs):** EVs are produced by the intestinal microbiota and are membrane-bound vesicles, lipid bilayers containing proteins, lipids, nucleic acids, and metabolites [47]. EVs are dependent upon the type of bacteria. Gram-negative bacteria produce larger (20–200 nm) outer membrane vesicles (OMV) and are more intricate in structure than those released by Gram-positive bacteria [48]. *Lactobacillus* spp. are types of bacteria which form extracellular vesicles that have characteristics and properties that aid in the prevention of the formation of tumors [49]. With regard to colorectal cancer, specific *Lactobacillus* species—*L. casei*, *L. rhamnosus GG*, and *L. acidophilus*—have been studied to reveal their therapeutic anti-cancer effects, probably through the help of EVs due to the DNA and proteins encompassing these vesicles [50].

## 5. Role of Postbiotics in Gut Microbiotic Health and Cancer Microbiotic Health

Studies have shown that in disease states including cancer, there is a decrease in beneficial bacteria—*Bifidobacterium*, *Lactobacillus*, and *Bacteroides*—and an increase in proinflammatory bacteria—*Escherichia coli*, and *Clostridium difficile* [29]. In the development of colorectal cancer (CRC), an abundance of toxic, opportunistic bacteria such as *Bacteroides fragilis*, *Enterococcus faecalis*, and *Streptococcus gallolyticus* are found in patients. The intestinal microbiota aids in cancer progression by damaging the mucosal barrier, influencing the cell cycle of cancer cells, promoting DNA damage, inducing inflammatory reactions, and inducing gene mutations [51].

Various postbiotics can selectively induce apoptosis in CRC, inhibit cellular proliferation, growth, and migration, and modulate the immune system. They can also go beyond suppressing carcinogenic signaling pathways, maintaining intestinal epithelial integrity and having a synergistic effect with chemotherapy drugs [52].

Consequently, postbiotics and their bioactive derivatives have a plethora of benefits, such as therapeutic effects on gastrointestinal physiology, immunoregulating effects, and anticarcinogenic effects, as well as enhancing cancer therapies [7]. Overall, these metabolites have shown improvement in colon, gastric, hematologic, breast, and cervical cancers [53]. Clinically, postbiotics and their derivatives, paired with cancer therapies, result in the creation and maintenance of beneficial bacteria in the patients, aid in the recovery after cancer surgery, and help to prevent surgical infection after cancer surgery, thereby decreasing hospital stay [54]. Postbiotics have also been shown to prevent the side-effects of traditional cancer drugs such as vomiting and diarrhea.

### 5.1. Colorectal Cancer

The gut microbiota has implications in the development of tumors in the host [55]. Gastrointestinal cancers, such as colorectal cancer, are caused by dysregulation of the intestinal bacteria and the proliferation of bacteria such as *Helicobacter (H.) pylori*, *Streptococcus (S.) bovis*, and *Enterococcus* [56]. Dysbiosis of these pathogens induces tumor growth and influences the immune system through releasing toxins and the promotion of several pathways. With regard to gastric cancer, it was seen that a *Lactobacillus paracasei* GMNL-133 (SGMNL-133) isolate enhanced therapeutic efficacy [57]. This was seen through anticarcinogenic mechanisms such as intestinal microbiota proliferation, immunoregulation, decreased levels of inflammation, and the activation of antitumorigenic substances [58].

### 5.2. Breast Cancer

*Lactobacillus* and *Lactococcus* species are found in abundance in healthy breast tissue in comparison to tissues with breast cancer [59]. In a study conducted by Wasiak et al., the influence of lactic acid bacteria (LAB)-derived postbiotics on the growth, expansion and influence of the cell cycle of breast cancer cells was studied. In their findings, postbiotics were shown to trigger apoptosis, with little influence on normal cell survival. In addition, the cytotoxic effect of tamoxifen was heightened, leading to a reduction in proliferation, suggesting that these agents can be used in combination with synthetic drugs. [60].

### 5.3. Gastric Cancer

Gastric cancer has routinely been treated with chemotherapy. However, this avenue of treatment can lead to gastrointestinal dysfunction, which can consequently limit the medication dose, lead to treatment discontinuation, and create life-threatening risks [57]. Recently, interest has been directed at the anticancer characteristics of postbiotics in relation to gastric cancer. Treatment with lysate extracts of *L. paracasei* significantly reduced the potency of gastric cancer cells [61]. This sheds light on the role that microbes have in cancer physiology. It was found that the active segments of postbiotic compounds in the range between 50 and 100 kDa and >100 kDa had the most effective tumor-inhibitory properties [57].

### 5.4. Cervical Cancer

SCFAs and other postbiotic metabolites from *Lactobacillus* keep the vaginal pH low and thus help to keep pathogens involved in cervical cancer and carcinoma in situ [62]. Linoleic acid released from bacterial metabolism influences gene expression and the expression of growth factors which modulate cell proliferation, differentiation, and maintenance [63]. In addition, butyric acid has also been found to decrease cervical cancer growth by interfering with cancer cell metabolism and through the promotion of cell cycle arrest. Butyric acid functions as an inhibitor of histone deacetylase, thus limiting the severity of cancer progression [64].

### 5.5. Leukemia

Postbiotics have shown anticancer potential with regard to leukemia [65]. The inhibition of the human leukemia cell line HL-60 was seen after a kimchi extract containing postbiotics was applied. It was found that the increased level of ornithine in kimchi was effective in suppressing the growth of cancer cells [66].

LAB displayed anti-tumor growth through the activation of apoptosis, cell cycle arrest, and antimutagenic effects. LAB-regulated immune reactions occur through tryptophan metabolism and the antioxidant properties of folic acid [67]. In an experiment, six strains of *Lactobacillus plantarum* were applied to different human leukemia cell lines [68]. It was found that bacteria metabolites expressed exclusive time- and dose dependent cytotoxic effects on these cells without impacting normal cells.

## 6. Mechanism of Action of Postbiotics in Cancer

Postbiotics influence a variety of physiological processes in the host, leading to health-promoting outcomes [69]. In 2024, the very first report showcasing the beneficial effects of heat killed *Lacticaseibacillus paracasei* MCC1849 on human immune cells was published [70]. This randomized, double-blind, placebo-controlled, parallel-group study involved 100 healthy adults randomly assigned to either the MCC1849 or placebo group. Participants consumed a test powder containing 5 × 10^10^ MCC1849 cells or a placebo powder for 4 weeks. The results revealed that the ingestion of MCC1849 activated peripheral dendritic cells (DCs) and maintained the expression levels of IFN-α, β, and γ under infection-like conditions.

Microbial-derived metabolites exert anti-inflammatory effects by generating anti-inflammatory molecules. Bioactive compounds secreted by *L. acidophilus* and *L. rhamnosus GG* (LGG) reduced the levels of MMP-9, decreased CD147 expression, and increased TIMP-1 expression in an inflammatory macrophage model in vitro [71]. Additionally, peptides from the 3–10 kDa IP fraction of *S. thermophilus* demonstrated anti-inflammatory properties by modulating proinflammatory mediators like IL-1β in LPS-stimulated THP1 macrophages [72].

Postbiotics are able to enhance the integrity of the intestinal barrier, decreasing the passage of harmful substances and potential carcinogens into the systemic circulation. Indeed, postbiotics have the potential to influence the production of mucus by stimulating goblet cells, specialized cells responsible for mucus secretion in the gut [73]. Moreover, after ethanol exposure, oral tributyrin preserved the expression of E. Cadherin and ZO-1, essential for the integrity of the small intestinal barrier. Tributyrin also reduced endotoxemia, accompanied by the promotion of immune tolerance in DCs within the small intestinal lamina propria and the nonactivation of intestinal microvascular endothelial cells [74].

**Figure 2:**
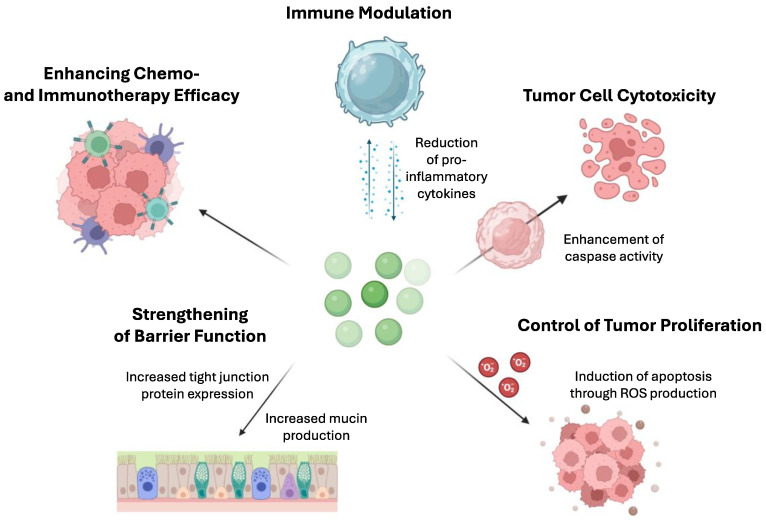
Mechanisms by which postbiotics enhance the effectiveness of chemotherapy and immunotherapy in cancer care. This figure illustrates several actions of postbiotics: (1) increasing tumor cell cytotoxicity by enhancing caspase activity, thereby promoting apoptosis in cancer cells; (2) immune modulation by reducing proinflammatory cytokines, contributing to a more balanced immune response; (3) controlling tumor proliferation by inducing apoptosis through the production of reactive oxygen species (ROS); (4) strengthening barrier function by increasing mucin production and tight junction protein expression, reducing the passage of potential carcinogens into the systemic circulation; and (5) increasing the effectiveness of standard chemotherapy and immunotherapy by sensitizing cancer cells to these treatments or by regulating immune responses to enhance antitumor activity. These complex mechanisms highlight the potential of postbiotics as adjuncts in cancer therapies.

Finally, postbiotics influence host metabolism by modulating gut microbiota composition and activity. They can selectively stimulate the growth of beneficial bacteria in the gut, leading to the production of short-chain fatty acids (SCFAs), such as acetate, propionate, and butyrate [75], which serve as energy sources for intestinal epithelial cells and can also be absorbed into systemic circulation, where they exert systemic effects on metabolism [76]. Butyrate, in particular, has been shown to enhance mitochondrial function and promote fatty acid oxidation in peripheral tissues, improving lipid metabolism. Additionally, SCFAs influence the secretion of gut hormones involved in regulating appetite and glucose metabolism, such as peptide YY (PYY) and glucagon-like peptide 1 (GLP-1), contributing to glucose homeostasis [77,78]. Aside from these general mechanisms, postbiotics demonstrate specific actions directly affecting cancer cells as demonstrated in Figure 2.

Nowak and colleagues investigated the antiproliferative effects of post-fermentation media (PFM) and cell extracts (CEs) from various strains of lactic acid bacteria on Caco-2 and HeLa cells. They found that both PFM and CEs induced oxidative stress in Caco-2 cells by increasing hydrogen peroxide production and ROS levels. Additionally, PFM from *L. plantarum* 0991 and *L. brevis* 0983 triggered apoptosis, as evidenced by the activity of caspases 3/7 and 9, indicating the potential involvement of the mitochondrial signaling pathway, possibly leading to late apoptosis or necrosis [79]. Similar results were also observed in several cancer cell lines, including the human breast cancer cells MCF-7, the colorectal cancer cells HT-29, the liver cancer cells Hep-G2, and the leukemia cells HL60 and K562, treated with postbiotics produced by Lactobacillus sp. La1, La2, and *Lactobacillus plantarum* [68,80].

Postbiotics reshape the tumor microenvironment by modulating immune responses. A randomized, double-blind, placebo-controlled trial in China tested the effect of JK5G postbiotics in non-small-cell lung cancer patients [81]. JK5G administration led to a significant decrease in the proinflammatory markers TNF-a, IL-2, and *C*-reactive protein. Notably, there were significant increases in CD3+ and CD4+ T cells and the CD4/CD8 ratio in the peripheral blood of JK5G group patients. Moreover, the JK5G group exhibited a superior quality of life and nutritional status, along with reduced depression symptoms, a lower incidence of anemia, a decreased lymphocyte count, reduced appetite, nausea, and asthenia compared to the control group. Furthermore, JK5G supplementation mitigated the gut microbiota imbalance by increasing the levels of beneficial bacteria such as *Faecalibacterium* and *Ruminococcaceae* and reducing the levels of *Escherichia-Shigella*.

Finally, microbial-derived products improve the effectiveness of standard chemotherapy and immunotherapy by sensitizing cancer cells to these treatments or by regulating immune responses to enhance antitumor activity. Extracellular vesicles derived from LGG improved anti-PD-1 immunotherapy efficacy against colorectal cancer by increasing the CD8+ T/CD4+ T cell ratio in mesenteric lymph nodes and enhancing the ratio of MHC II+ DC cells, CD4+ T cells, and CD8+ T cells in tumor tissues. Moreover, significant changes occurred in the levels of serum metabolites linked to the microbiota, contributing to antitumor effects [82]. Huang HL et al. tested a potential nanoparticle formulation encapsulating the *Lacticaseibacillus paracasei* GMNL-133 isolate. This innovative approach safeguarded SGMNL-133 from gastric acid degradation, facilitated its passage through the mucus layer, and promoted interaction with gastric cancer cells. Moreover, in vivo experiments demonstrated that encapsulating SGMNL-133 in nanoparticles significantly enhanced its efficacy in treating orthotopic gastric tumors while concurrently reducing tissue inflammation levels [57].

## 7. Prebiotics, Postbiotics, and Purified Macromolecules for Cancer Care—Preclinical and Clinical Studies

Recently, the research interest has been focused on the action of the gut microbiota and its metabolites and, therefore, its potential benefits in preventing cancer, improving oncological treatments and preventing their side effects.

A wide range of studies have investigated the effects of postbiotics on cell lines and mice in preclinical studies; indeed, according to the definition of postbiotics, inactivated microbes, their fragments, and their molecules have been studied in order to identify possible benefits [83]. In particular, evidence has led investigators to focus on molecules such as lipotheicoic acid (LTA), lypopopysaccharides (LPS), and short-chain fatty acids (SCFAs) inducing antitumoral effects [84]. Pieces of evidence provided by several authors are summarized in Table 2.

Concerning clinical trials in human beings, postbiotics as strictly defined in the literature have been tested in only a few studies. However, if we consider microbially purified molecules derived from inactivated bacteria, these have been more extensively used in interventional studies in oncological patients. In this regard, vitamin K molecules are the only postbiotics largely found in the gut rather than in the nutritional intake and used in oncological clinical trials, although this has led to mixed results. Indeed, results in prostatic cancer trials are largely disappointing [93], but promising results have been shown in hepatocellular cancer studies [94]. The latter study was a randomized phase II trial, performed on 38 patients assuming sorafenib, and showed better progression-free survival (PFS) and objective response rate (ORR) in patients assuming phylloquinone and menoquinone, concomitantly. However, the overall survival (OS) and disease control rate remained not significative and post hoc analyses suggested that the patients who really benefited from the supplementation were only those belonging to the subgroup with a radiological response.

Generally, clinical studies have focused on the administration of prebiotics and monitoring fecal microbiome metabolite levels rather than the direct administration of postbiotics. Nevertheless, several studies have reported heterogeneous results on the role of short-chain fatty acids (SCFAs). Noteworthily, the LIBRE trial investigated the presence of SCFAs in women bearing BRCA 1/2 mutations, with or without a previous diagnosis of breast cancer, describing an improvement in the enteric mucosal barrier integrity [95] in patients with higher fecal quantities. Also, the link between colorectal cancer (CRC) and SCFAs was investigated in several observational studies, showing significant higher proportions for acetic acid (AA) [96,97], propionic acid, and butyric acid (BA) [97] in healthy patients rather than in affected patients, although this did not confirm a causal relationship. Analogously, some authors have also deepened the relationship between SCFAs and the CRC risk by comparing higher-risk subjects (with history of colorectal adenomas) with lower-risk subjects (apparently healthy patients), detecting higher fecal proportions of BA [98,99,100,101], PA, and AA [99] in the latter. However, several trials with negative results have created inhomogeneities in results and doubts for SCFAs’ role in CRC [68] and disease risk [94,100,101]. A metanalysis aimed to address the uncertainties on the matter and confirmed statistically significant results for SCFAs as a group effect, but not for the single molecules (AA, BA, and PA), both for predicting CRC risk and incidence [102]. Noteworthily, this metanalysis confirmed higher levels of BA and AA, but not PA, in healthy patients than in patients affected by CRC. However, it has to be mentioned that this metanalysis suffered from high heterogeneity (I2 50–90%).

Concerning interventional studies, the RIBOGUT trial showed how the oral riboflavin supplementation could lead to increasing levels of BA, but not other SCFAs, suggesting an interaction with the gut microbiome and the incrementation of postbiotics [103]. Similarly, evidence for the production of AA and PA derives from the assumption of legume kernel fibers like blue lupins, suggesting a role in the production of intestinal postbiotics [104]. Importantly, some interventional studies have also investigated the use of prebiotics for increasing the quantities of intestinal postbiotics after surgery or during chemotherapy treatment.

In this regard, an earlier study analyzed CRC patient’s feces after oncological surgery, finding lower quantities of SFCAs [105], defining an area of intervention for the administration of oral fibers aimed at increasing the level of SCFAs. Moreover, evidence of an SCFA increment, faster recovery, and improvements in the immunologic indices was described for patients undergoing oncological surgery [106]. Concerning chemotherapeutic toxicities, a Japanese study showed a link between the administration of synbiotics (a combination of prebiotics and probiotics), increasing proportions of fecal SFCAs, and mitigation of the toxicities in Asiatic patients [107]. Notably, they observed a significant reduction in lymphopenia, diarrhea, and febrile neutropenia induced by chemotherapies [103].

The main characteristics of the most relevant studies explored in this section are summarized in Table 3.

Other prebiotics did not add new information to this argument. One of the most relevant examples is inulin supplementation, a complex carbohydrate that is not fully digestible, which is renowned to be a modulator of SCFA production. It has been used in some interventional studies, demonstrating important benefits in animal models [108], but its role in clinical trials remains controversial [109].

Over time, the landscape of gut microbiota metabolites has expanded from the classical postbiotics to several metabolic fingerprints based on volatile organic compounds and their relationship with CRC, with promising potential in the near future [110].

**Table 3:** Clinical trials involving purified metabolites, postbiotic derivates, and prebiotics for postbiotic measurable production.

	Intervention	Type of Study	Type of Cancer	Median Age (IQR)	Sample Size (n. Events)	Median Follow Up	Antitumoral Benefit
Boutron-Ruault MC et al., 2005 [100]	Supplementation with s-FOS	Interventional prospective study	- Small adenomas - Large adenomas - Healthy controls	61 (8)	74	-	Higher fecal butyrate concentration in the adenoma group after the 3-month administration of sc-FOS
Chen HM et al., 2013 [99]	None	Cross-sectional observational study	Patients with a resected AP vs. healthy controls	58 (11)	391	-	Lower SCFA levels were found in the AP group Clostridium, Roseburia and Eubacterium spp were retrieved at higher levels in the healthy controls Enterococcus and Streptococcus spp. were more highly represented in the AP group
Motoori M et al., 2017 [107]	Synbiotic supplementation 10 days after chemotherapy	Phase II, randomized open-label study	Esophageal cancer	-	61	-	Decrease in toxicity incidence (nausea, diarrea and febrile neutropenia)
Hoyt M et al., 2019 [93]	Menokinones, phylloquinone	post hoc Observational study	Prostate cancer	63 (6)	28,356 (2978)	11.3 months	No benefits as risk-reducing factor
Niccolai E et al., 2019 [96]	None	Cross-sectional, controlled, nonrandomized, observational study	Colorectal cancer vs. AP and healthy controls	CRC: 80 (13) AP: 46 (8)	60	-	CRC patients showed increased levels of butyric isobutyric, valeric and isovaleric acid, whereas the levels of acetic acid were reduced
Xie X et al., 2019 [106]	Supplementation with 30 mg/d of prebiotics (fiber) aiming to raise intestinal SCFAs	Interventional prospective study	Colon cancer development	60 (9)	135	-	Preoperative period: higher levels of IgM, IgG and transferrin Postoperative period: higher levels of IgA, IgG, CD8+ Cells, B-cell lymphocytes
Ocvirk. S et al., 2020 [98]	None	Cross-sectional observation study	Apparently healthy patients	51 (8)	53 (AN: 32 RA: 21)	-	AN ate more fatty and caloric food than RA In AN, 16 out of 32 patients had colic adenomatous polypolsis, whereas none of RA developed polyposis. Stools from RA were more enriched by SCFAs than AN stools.
Haruna Y et al., 2021 [94]	Vitamin K + sorafenib vs. sorafenib alone	Phase 2, randomized, open-label study	Hepatocarcinoma	72 (8)	44 (44)	70 months	Benefit for ORR (27.3% vs. 4.5%, *p* = 0.039) PFS (HR = 0.59, *p* = 0.12) No benefit for OS (HR = 0.59, *p* = 0.12)
Seethaler B et al., 2022 [95]	Increase in SCFA production through diet and physical activity	Phase 2, randomized, controlled, open label study	BRCA-mutated patients	44 (2)	260 women	-	Increase in level of fecal SCFAs produced Decrease in intestinal permeability mediated by SCFAs
Motoori M et al., 2017 [111]	Synbiotic supplementation in addition to enteral nutrition and prophylactic antibiotics during neoadjuvant chemotherapy	Phase II, randomized open-label multicenter study	Esophageal cancer	-	81	-	Signficiant decrease in grade 3 and grade 4 toxicity incidence (nausea, diarrea and neutropenia) but not febriel neutropenia (*p* = 0.088)
Liu L et al., 2023 [103]	Supplementation with either 50 or 100 mg/d of Riboflavin for 2 weeks	Interventional prospective study	Colon cancer development	31 (11)	105	28 days	Higher fecal butyrate level with riboflavin supplemantetion regardless of the given dose

AN: Alaska-native patients, AP: adenomatous polyp, RA: rural African patients, s-FOS: short-chain fructo-oligosaccharides.

## 8. Conclusions and Future Perspectives

The role of postbiotics in cancer care is mainly associated with the function of the host immune system and the modulation of inflammatory responses. In this sense, postbiotics integration may have a role both in carcinogenesis prevention and in cancer care [7]. Up to now, postbiotics have not been directly evaluated in phase II and III trials in cancer care, with the only exception being vitamin K molecules [93,94]. Meanwhile, the levels of postbiotics such as SFCAs have been evaluated indirectly in clinical interventional studies testing the administration of prebiotics and probiotics, such as in the RIBOGUT trial [103]. Microbiota-derived SFCAs have shown promising and synergistic activity in anticancer treatment and a role in favoring immune responses [112].

Postbiotics have some advantages over probiotics. First of all, probiotics are made from live microorganims which require the concomitant presence of prebiotic fibers in the gut microbiome to be more effective. Meanwhile, postbiotics do not require the presence of prebiotics. Moreover, probiotics require ideal conditions in terms of temperature, moisture, and oxygen tension in order to be kept alive, while there are no viability issues associated with postbiotics [28]. Furthermore, as long as they contain live microorganisms, probiotics may present some degree of risk when used in more vulnerable populations. Meanwhile, postbiotics are unique strains or nonliving microbes and their use is not associated with a risk of bacterial infection [113]. The lack of consistent clinical evidence supporting postbiotics’ efficacy is due to their poor bioavailability, short half-life, and fluctuating levels [114]. Indeed, postbiotics’ levels are strictly dependent on the gut microbiota’s composition, which may vary between individuals and can be altered under different physiological and pathological conditions. Therefore, interindividual variability, the need for industrial purification, and regulatory affairs may be among the reasons why postbiotics have not been widely tested and developed so far by pharma companies. In this direction, together with more efforts in order to facilitate the production and use of postbiotics, the development of synbiotics is of increasing interest. Synbiotics are a mixture of prebiotics and probiotics, conjugating the activity of live microorganisms and nondigestible fibers, and are expected to have a more homogeneous bioavailability than postbiotics.

## Figures and Tables

**Table 1:** This is a comparative table outlining the features of prebiotics, probiotics, and postbiotics.

Characteristics	Prebiotics	Probiotics	Postbiotics
Origin	Nondigestible fibers	Live microorganisms	Inanimate, dead or inactivated microorganisms
Sources	Vegetables and fruits	Yogurt, kefir, kimchi, miso, tempeh	Kefir, kombucha, yogurt, miso, tempeh, kimchi, butter
Primary Role	Provide nourishment to probiotics	Improve digestion, strengthen the immune system and positively influence mental health	Offer similar benefits of probiotic without the introduction of live microorganisms

**Table 2:** Postbiotics used in preclinical studies.

	Postbiotic Used	Type of Study	Bacterial Source	Type of Cancer	Up/Down	Antitumor Activity
Li et al., 2019 [85]	LPS	In vitro	*Helicobacter pylori*	Gastric cancer cell lines (SGC7901, BGC823, others)	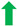	Proliferation
					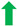	Migration
Arabzadeh et al., 2016 [86]	LTA LPS	In vitro	-	Ovarian cancer cell (*SKOV-3 cell line*)	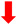	Cell viability
					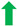	Inflammation
					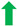	Cell invasion
					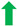	Wnt5A–ROR2 complex
Deepak et al., 2016 [87]	EPS	In vitro	*Lactobacillus acidophilus*	Colon cancer cells	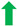	TIMP-3
					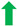	HO-1
					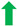	HIF-2α
					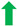	PAI-1
					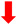	VEGF
					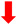	HIF-1α
Hattar et al., 2017 [88]	LTA	In vitro	*Staphylococcus aureus*	Colorectal cancer (HCT-116 cell line)	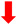	Apoptosis
					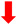	Adhesion
					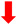	Migration
Xie et al., 2012 [89]	LTA + 5-fluorouracil	In vivo	*Bifidobacterium*	Hepatoma-22 cells inoculated in mice	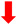	Tumor growth
					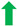	T lymphocyte proliferation
					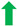	IFN-gamma regulatory
					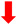	T-cells
					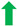	TIM-3
					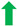	FOXP3
Sadeghi et al., 2020 [90]	Beta-Glucan	In vitro	*Candida albicans*	Lung cancer cells	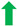	SOX2
					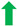	OCT4
Luo et al., 2019 [91]	Sodium Butyrate	In vitro	-	Colorectal cancer cells (HCT-116 cell line)	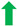	Autophagy
					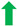	Autolysosomes
					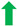	AMP kinase
					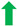	LKB1
Watkins et al., 1999 [92]	Sodium Butyrate	In vitro	-	Hep G2 cells	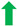	Histone H4 Acetylation
					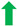	DNA Fragmentation

EPS: exopolysaccharides; FOXP3: forkhead box P3; HIF-1α: hypoxia-inducible factor-1α; HIF-2α: hypoxia-inducible factor-2α; HO-1: hemeoxygenase-1; IFN: interferon; LKB1: liver kinase B1; LPS: lipopolysaccharides; LTA: lipoteichoic acid; OCT4: octamer-binding transcription factor 4; PAI-1: plasminogen activator inhibitor-1; SOX2: sex determining region Y-box 2; TIM-3: T-cell immunoglobulin and mucin domain 3; TIMP-3: tissue inhibitor of metalloproteinases-3; VEGF: vascular endothelial growth factor; Wnt5A: wingless-related MMTV integration site; ROR2: Receptor tyrosine kinase-like orphan receptor 2. Green arrow—upregulation, red arrow—downregulation.
